# Suboptimal Omnidirectional Wheel Design and Implementation

**DOI:** 10.3390/s21030865

**Published:** 2021-01-28

**Authors:** Jordi Palacín, David Martínez, Elena Rubies, Eduard Clotet

**Affiliations:** Laboratory of Robotics, Universitat de Lleida, Jaume II 69, 25001 Lleida, Spain; david.martinez@udl.cat (D.M.); helenarubies@gmail.com (E.R.); eduard.clotet@udl.cat (E.C.)

**Keywords:** mobile robot, omnidirectional motion, vibration measurement

## Abstract

The optimal design of an omnidirectional wheel is usually focused on the minimization of the gap between the free rollers of the wheel in order to minimize contact discontinuities with the floor in order to minimize the generation of vibrations. However, in practice, a fast, tall, and heavy-weighted mobile robot using optimal omnidirectional wheels may also need a suspension system in order to reduce the presence of vibrations and oscillations in the upper part of the mobile robot. This paper empirically evaluates whether a heavy-weighted omnidirectional mobile robot can take advantage of its passive suspension system in order to also use non-optimal or suboptimal omnidirectional wheels with a non-optimized inner gap. The main comparative advantages of the proposed suboptimal omnidirectional wheel are its low manufacturing cost and the possibility of taking advantage of the gap to operate outdoors. The experimental part of this paper compares the vibrations generated by the motion system of a versatile mobile robot using optimal and suboptimal omnidirectional wheels. The final conclusion is that a suboptimal wheel with a large gap produces comparable on-board vibration patterns while maintaining the traction and increasing the grip on non-perfect planar surfaces.

## 1. Introduction

Cooperation between humans, mobile robots, robots, and other devices will change the development of many essential repetitive task such as packaging, delivering, cleaning, maintenance, and surveillance. On the one hand, the automatic development of such cooperative tasks requires the use of human compatible devices in order to avoid collisions or actions that can harm people, mainly using laser range sensors [1,2], depth cameras [3,4], or surveillance systems [5]. On the other hand, the automatic development of cooperative tasks requires the use of human compatible devices moving like people in common spaces designed for people, mainly using legs [6], omnidirectional wheels [7], or hybrid legs [8,9].

The development of additive manufacturing and 3D printing is currently fostering the development and implementation of many robotic applications with a reasonable cost and maintenance. For example, Stroud et al. [10] designed and implemented a teen-sized humanoid soccer robot based on 3D printing, Ćurković et al. [11] proposed a legged walking robot based on 3D printing, Chavdarov et al. [12] proposed the development of a walking robot using 3D printing, Joyee et al. [13] proposed the development of a soft robot based on 3D printing, and Rubies et al. [14] proposed the implementation of a miniature compact omnidirectional wheel using 3D printing.

The development of mobile robots or mobile platforms for common spaces designed for people usually have space and motion limitations. Mobile robots using two independent driving wheels have mobility restrictions to one direction and are not able to perform transversal displacements. Alternatively, omnidirectional mobile robots usually have four mecanum wheels [15] or three or four synchro-drive wheels [16] with individual control of the orientation and rotation speed. These omnidirectional configurations can perform transversal displacements but have limitations when performing combined or fast displacements [17]. These motion limitations do not exist when using three or four optimal omnidirectional wheels [18], but then the kinematic control becomes more complex, the wheel assembly requires the use of expensive machinery, and the motion system is prone to the generation of vibrations due to wheel discontinuities [19,20,21].

The development of this paper is based on the humanoid-size Assistant Personal Robot (APR) presented in Clotet et al. [22], which uses a three-wheel omnidirectional motion system based on the implementation of an optimal omnidirectional wheel [18]. The basic idea of the optimal omnidirectional wheel is the minimization of the gap between the free rollers that proved the omnidirectional capabilities. The advantage of this configuration is the implementation of the omnidirectional capabilities with only three omnidirectional wheels and three motors. However, this optimal configuration has the drawbacks of the high force applied to the thinnest part of the pieces that support of the free rollers, the large number of parts required to assemble a wheel, the high complexity and cost of the manufacturing of these pieces in durable aluminum using computer numerical control (CNC), and the generation of vibrations due to the rotation of the omnidirectional wheel. In the case of the APR, the tall frame of the mobile robot amplified the vibrations generated by the rotation of the omnidirectional wheels, causing the robot head to vibrate ostensibly and limiting the practical application of this mobile robot.

Recently, Moreno et al. [21] improved the APR design by the inclusion of a passive suspension system optimized to reduce the transmission of vibrations from the omnidirectional wheels to the head of the mobile robot. This passive suspension, based on the use of rubber dumpers and spring shock absorbers, reduced the generation and transmission of vibrations and finally fostered the development of mobile robot applications based on the APR design concept. 

This paper proposes taking full advantage of the passive suspension system currently included in APR mobile robots in order to evaluate the possibility of using simple and cheap omnidirectional wheels not focused on the minimization of the gap between the free rollers of the omnidirectional wheel. This non-optimal or suboptimal design and implementation is proposed to take advance of standard mass-produced pieces and cheap 3D printing in order to reduce the expensive manufacturing cost required by the optimal omnidirectional wheels used currently in APR mobile robots. However, it must be expected that the use of a suboptimal omnidirectional wheel will generate higher vibrations during the rotations, so the question that arises is whether the existing passive suspension system will be able to also reduce the higher vibrations generated by a suboptimal omnidirectional wheel. If the answer to this question is no, then the use of suboptimal omnidirectional wheels in APR mobile robots will require further improvements such as the implementation of active control strategies focused on the problem originated by the impact of the suboptimal omnidirectional wheel with the floor, as is done, for example, in hexapod wheel-legged robots [9]. However, this paper proves that the answer to this question is yes, the existing passive suspension system is able to reduce the transmission of vibrations from the wheel to the head of a tall APR mobile robot. Therefore, the mobile robot will benefit from using much cheaper omnidirectional wheels and even get outdoor capabilities thanks to the additional grip provided by the gap between the free rollers of the omnidirectional wheels. 

The paper is structured as follows: Section 2 presents the materials and methods used. Section 3 is a verification of the results of Moreno et al. [7] and compares the vibrations generated by an APR mobile robot using an omnidirectional motion system with and without a passive suspension system. Section 4 presents the optimal omnidirectional wheel used up until now in APR mobile robots and the proposed suboptimal omnidirectional wheel design, implementation using low-cost pieces, and low-cost 3D printing techniques. Section 5 compares the vibrations generated directly by one optimal omnidirectional wheel and one suboptimal omnidirectional wheel with a support piece 3D printed either as soft (partially empty) or hard (almost solid). Section 6 compares the vibration performances of two different APR mobile robots using the new suboptimal omnidirectional wheels proposed in this paper. Finally, Section 7 outlines the discussion and conclusions of the paper. The raw accelerometer and motor speed data used in the experimental part of this paper are available to download as supplementary data files in plain text format (.txt).

## 2. Materials and Methods

### 2.1. Mobile Robot Prototypes

**Figure 1:**
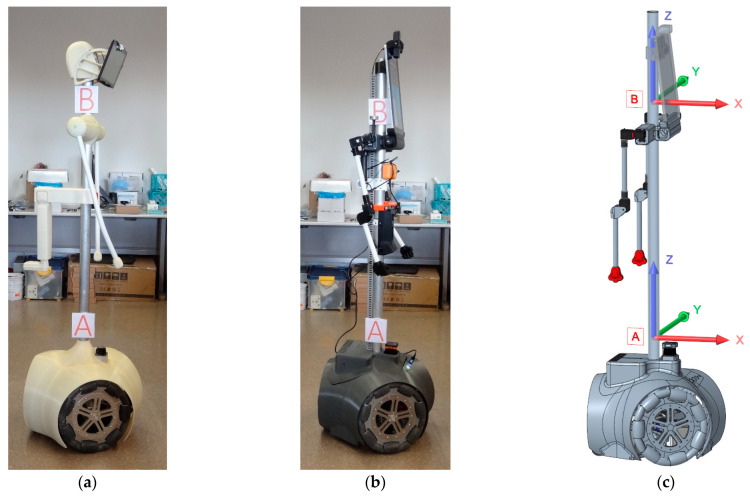
Assistant Personal Robots (APRs): (**a**) APR-01, first prototype version of the APR robot, including an omnidirectional motion system based on three omnidirectional wheels without a suspension system; (**b**) APR-02, improved prototype version, including the same omnidirectional motion system with an optimized suspension system; and (**c**) APR-02 CAD design, including a representation of the X, Y, and Z axes used to reference the measure of vibrations.

The main experimental materials used in this paper were two humanoid mobile robot prototypes. Figure 1a shows an image of the first APR prototype version, named APR-01 and described in Clotet et al. [22]. This tall mobile robot is based on an omnidirectional motion system based on three omnidirectional wheels and two arms. This prototype is based on a rigid and tall structure that transmits and amplifies the vibration generated by the rotation of the wheels. The existence of these unexpected vibrations limits the operational velocity and the practical application of this mobile robot. Figure 1b shows an image of the second APR prototype version, named APR-02, which was improved with the inclusion of a passive suspension system optimized to reduce the transmission of vibrations from the wheels to the dynamic internal structure of the mobile robot. Finally, Figure 1c shows a Computer Aided Design (CAD) of the APR-02 mobile robot, including the reference system of the X, Y, and Z axes used in this paper to reference the measure of vibrations.

**Figure 2:**
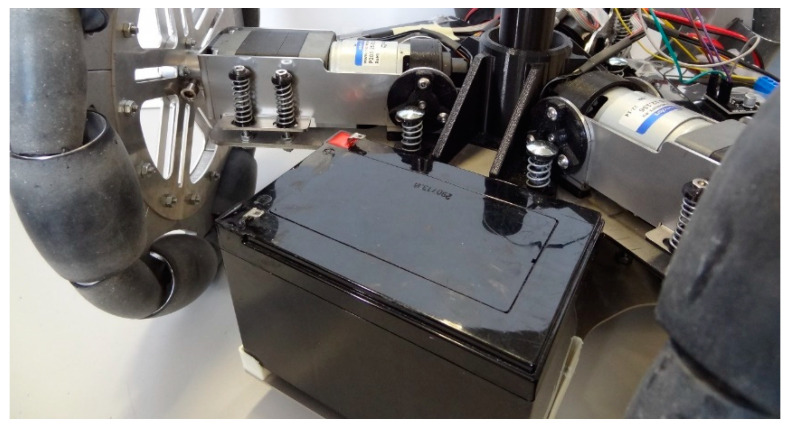
Detail of the passive suspension implemented in the APR-02 mobile robot prototype.

Figure 2 shows a closed illustrative detail of the optimized passive suspension system already implemented in the APR-02 mobile robot, which is based on the use of rubber dumpers and spring shock absorbers. This optimized passive suspension is fully described in Moreno et al. [21]. The Direct Current (DC) motor that supports the omnidirectional wheel has a rubber damper at the connection between the motor and its support structure. This support structure is then connected to the chassis of the mobile robot with four spring shock absorbers and a soft pivoting joint. This chassis supports the electronics, the body of the mobile robot, and a secondary chassis that supports the batteries and the central tower structure of the mobile robot. The connection between the chassis and the secondary chassis is provided by six spring shock absorbers. This configuration uses the weight of the three internal batteries and the central tower in order to create an effective spring-mass system that minimizes the existence of vibrations in the head of this tall mobile robot. The development of this optimized passive suspension system fostered the development of applications that take advantage of the mobility achieved by the omnidirectional motion system of the APR-02 mobile robot: In [23] this mobile robot was proposed for early gas detection and location, in [24] this mobile robot was used as a walk-helper tool, and in [25] this mobile robot was used to evaluate the use of a fixed push-broom 2D Light Detection and Ranging (LIDAR) for Simultaneous Location and Mapping (SLAM).

### 2.2. 3D Printer

The 3D printer used in this paper was a Printersys 325 fused filament fabrication (FFF) open-source 3D printer manufactured by Printersys (Algayón, Huesca, Spain). This FFF 3D printer has a printing area of 250 × 250 × 310 mm and a direct extruder with a single 0.4 mm nozzle, uses filament of Ø 1.75 mm, has a heated platform and a mobile Z-axis, and has coreXY kinematics with the X-axis and Y-axis motors fixed to the structure. The printing speed used in this 3D printer was 80 mm/s. The slicer used to convert from the Standard Triangle Language (STL) files used in CAD in to the gcode Computer Aided Manufacturing (CAM) files required by the printer was simplify3D 4.1.1 (Cincinnati, Ohio).

### 2.3. Measurement Module

The measurement module used to gather the experimental data analyzed in this paper is based on an ARM Cortex−M4 processor configured to directly control the power applied to the brushed direct current (DC) motors of the APR robots during the experiments, directly read the rotating speed of the motors using its original Hall-effect encoders, and measure the vibrations generated during the experiment.

The microcontroller used was a STM32F407VGT6, which has a Static Random Access Memory (SRAM) of 196 Kbytes, 1 Mbyte flash memory, several timers, several Pulse Width Modulation (PWM) generators, and several input capture interruptions. The brushed DC motors are controlled with the VNH5019A-E H-bridge motor driver designed for harsh automotive environments. This H-bridge is an updated version of the VNH2SP30-E originally used in APR robots. This H-bridge motor provides motor direction control, brake condition, PWM control up to 20 kHz, current monitoring, and an operation range of up to 41 V and 30 A. The low-cost brushed DC motors of APR robots have a magnetic rotary encoder based on a six-pole magnet and two Hall-effect sensors (U18 sensor from Unisonic Technologies CO.LTD) located 90° out of phase to each other, generating three pulses per motor turn. The rotation speed of the motor is deduced by measuring the elapsed time between two pulses. This measurement is performed with timer 2 configured as an input capture. This timer is a 32−bit clock configured to operate with an internal clock of 84 MHz. These configurations automatically provide a clock counter value when detecting a rising edge transition in the input signal. Then a routine computes the difference between two consecutive counter values and provides an updated estimate of the rotating speed of the motor three times per motor turn. The vibrations are measured with an onboard LIS3DSH accelerometer, an ultra-low-power high-performance three-axis linear accelerometer with a serial port interface (SPI) communication already used as onboard sensor on APR robots. This accelerometer has a dynamic measurement range selectable between ±2 and ±16 g, a sensitivity between 0.06 and 0.73 mg/digit, and a sampling rate of up to 1.6 kHz. The standard deviation of the acceleration measured in the X-, Y-, and Z-axes at zero speed were 8.04 mm/s^2^, 10.04 mm/s^2^, and 11.94 mm/s^2^, respectively. The configuration selected for the development of the experiments was a dynamic range of ±2 g, a sensitivity of 0.06 mg/digit, and a sampling rate of 1 kHz.

The measurement module is externally accessed through a universal asynchronous receiver–transmitter (UART) communication that can be connected to a Bluetooth module for wireless control. The measurement module can be configured to operate (1) as an autonomous device that directly controls the power applied to the brushed DC motors of the APR motors, reads the rotation speed of the motors, and reads the acceleration detected, or (2) as a slave device configured to read the acceleration detected when carried in an APR robot performing a movement or a task. The data gathered are stored as a text file in a USB flash-disk memory connected to one additional USB2.0 on-the-go (OTG) interface.

### 2.4. Boxplot Plotting Method

The graphical plotting representation used in this paper was the boxplot method introduced by J.W. Tukey in 1977 [26]. The boxplot method is widely used in the scientific literature as descriptive statistics for graphically depicting groups of data through their quartiles [27]. This plotting method is based on drawing a box from the first quartile (Q1) to the third quartile (Q3), a central mark indicating the median, and extending lines indicating variability outside the interquartile range (IQR), computed as the distance between the third and first quartile (IQR = Q3 − Q1), a value that represents 50% of the distributed data. The extreme minimum value is calculated as Q1 − 1.5·IQR and the extreme maximum value is calculated as Q3 + 1.5·IQR. The advantage of this representation is that it is not affected by the presence of outliers in the data analyzed.

## 3. Omnidirectional Motion System without and with a Passive Suspension System

The description, application, and advantages of the application of a suspension system in APR mobile robots was fully described in Moreno et al. [21]. As a summary, the conclusion was that the standard deviation of the vibration measured in the head of the mobile robot with a rigid body (without suspension), 1.3 g for the APR-01 robot, was reduced 16 times with the incorporation of a passive suspension system, 0.08 g for the APR-02 robot, reducing the mechanical fatigue of the pieces of the mobile robot. From an application point of view, this reduction totally eliminated the negative visual effect originated by the vibration of the head of the APR mobile robot and facilitated the development of new prototypes and applications.

Since their creation, mobile robots APR-01 (prototype without suspension, Figure 1a) and APR-02 (prototype with a passive suspension system, Figure 1b) have been updated and maintained. Therefore, the first experiment performed in this paper was a comparative revision of the vibrations measured in two parts of the tall central structure of the mobile robots. Figure 1 depicts the location of the measurement points used in this paper: A, at a floor height of 500 mm, and B, at a floor height of 1420 mm. In this experiment the measurement module was carefully attached at these measurement points, operating as a slave measurement device and sensing accelerations generated during a normal displacement of the mobile robots. Figure 1c shows a representation of the measurement axes used in this paper. 

**Figure 3:**
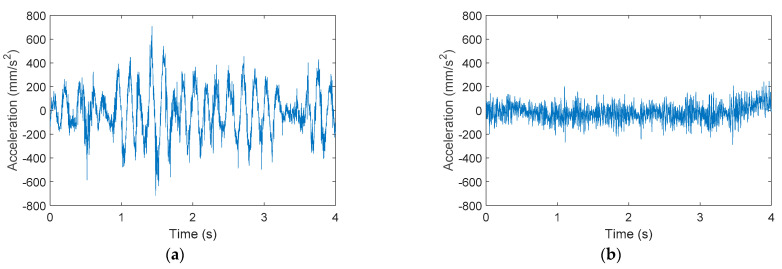
Raw acceleration data measured in location B: (**a**) gathered from APR-01 and (**b**) gathered from the APR-02 mobile robot, equipped with a suspension system.

Figure 3 shows the raw acceleration data measured at point B on the X-axis of APR-01 and APR-02. During this experiment both mobile robots moved forward at 0.40 m/s for 4 s. The X-axis, longitudinal to the forward movement, was the direction in which the vibrations exhibited maximum amplitude in both mobile robots. Figure 3a shows that the acceleration pattern gathered at the head of APR-01 (without a suspension system) had a fundamental frequency of 6.5 Hz and amplitudes around 400 mm/s^2^. Figure 3b shows that this fundamental frequency was not present in APR-02 thanks to the effect of its passive suspension system. In this case the amplitude of the acceleration was reduced to 150 mm/s^2^.

**Figure 4:**
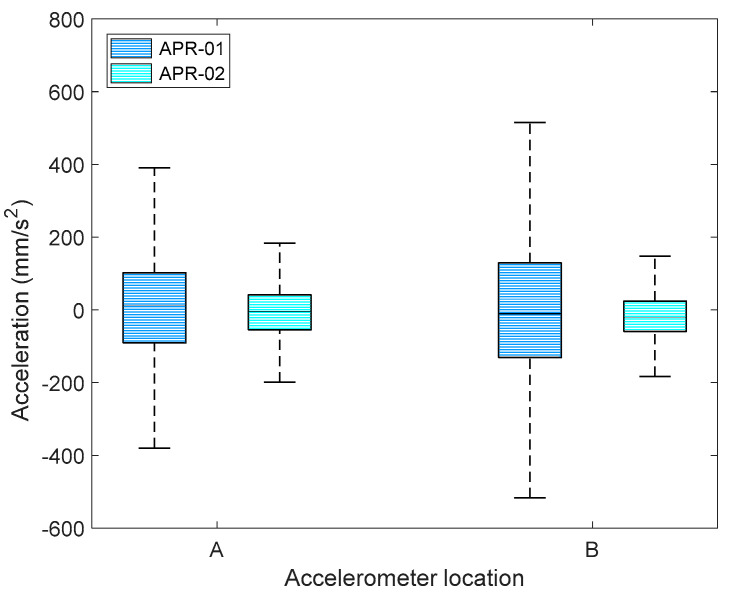
Representation of amplitude of the accelerations measured on the X-axis at locations A and B of the APR-01 and APR-02 mobile robot prototypes.

Figure 4 statistically summarizes the amplitudes of the accelerations measured on the X-axis at locations A (base) and B (head) of mobile robots APR-01 (without a suspension system) and APR-02 (with a suspension system). In both measurement points, the IQR of the accelerations measured in the APR-01 was reduced by 68% in APR02 because of the effect of the suspension system. These acceleration levels agreed with the results of Moreno et al. [21]. These measurements can be complemented with the visual impression of the accelerations measured. On the one hand, the high acceleration levels measured in the APR-01 robot (Figure 3a and Figure 4) produced a high mechanical stress in the pieces of the mobile robot and a highly disappointing visual impression; it seemed that the mobile robot was going to explode or disassemble at any moment. In practice, this poor visual impression forced the reduction of the operational velocity of APR-01 to 0.30 m/s. On the other hand, the low acceleration levels measured in ARP−02 were not visually appreciable even up to velocities of 0.99 m/s, although the nominal forward velocity used in all the applications was internally limited to 0.66 m/s in order to move more slowly than a person walking. This nominal forward velocity was achieved by applying a PWM of 60% to the DC motors of the mobile robots.

Therefore, the development of a suboptimal omnidirectional wheel and its application in APR-02 generated the question of whether the vibration pattern generated with this new implementation (APR-02 with a new suboptimal omnidirectional wheel and a suspension system) would behave like APR-01 (optimal omnidirectional wheel without a suspension system) or like APR-02 (optimal omnidirectional wheel with a suspension system).

## 4. Omnidirectional Wheel for a Three-Wheel Motion System

### 4.1. Original Optimal Omnidirectional Wheel

**Figure 5:**
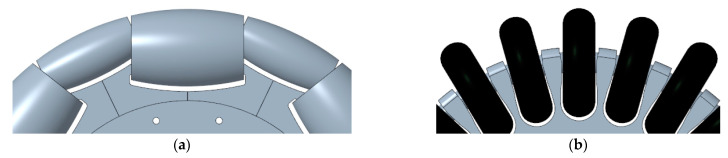
Omnidirectional wheel: (**a**) original optimal proposal implemented in the APR-01 and APR-02 robots and (**b**) new suboptimal alternative proposed.

The omnidirectional wheel created for use in the three-wheel omnidirectional system of APR mobile robots was described in Clotet et al. [22] and Moreno et al. [7]. In the context of this project, the concept of optimal omnidirectional wheel refers to the minimization of the gap between the free rollers used in the outer part of the wheel. Figure 5a shows a CAD representation of the omnidirectional wheel originally used in APR-01 and APR-02 in a three-wheel omnidirectional motion system. This omnidirectional wheel design provides the free transversal motion required in an omnidirectional motion system based on the use of three wheels. The optimal reduction of the gap between the rollers of the wheel is accomplished using a support piece that allows a small roller to fit inside a larger roller. In both cases the combined roof profile of the rollers is designed to coincide with the expected profile of a round wheel. This design enables the reduction in the gap that is only limited by the mechanical precision of the CNC machines used to create the rollers and the intricate inner support piece and the precision of the machines used to create the cover of the free rollers. In the omnidirectional wheels of APR-01 and APR-02 the value of this gap was only 2.5 mm but the manufacturing cost of these three wheels represented approximately 25% of the final cost of the complete mobile robot.

This optimal implementation also had other drawbacks: (1) the aluminum implementation of a complete omnidirectional wheel was very heavy, around 2.667 kg per wheel and thus 8.001 kg per mobile robot, and (2) the plain sliding profile of the covers of the rollers provided a poor grip. This plain cover profile is typically used in omnidirectional wheels because the use of friction bands may contribute to increasing the vibrations generated by the omnidirectional wheel. This typical plain profile may seem adequate for a flat and clean indoor floor but has strong practical limitations when the omnidirectional wheel has to pass over very small objects placed on the floor, such as wires that usually stop the motion or alter the direction of the motion of the omnidirectional wheel. In practice, this strong limitation to passing over small objects can have unexpected operational limitations because a mobile robot using this omnidirectional wheel can be easily blocked when trying to enter or exit the elevator in case of misalignment between the floor of the elevator and the floor or even when trying to pass over the expansion joint of a large building. 

### 4.2. Proposed Suboptimal Omnidirectional Wheel

The hypothesis of this paper is to take advantage of the passive suspension system included in an existing mobile robot prototype in order to overcome the problems generated by a non-optimal omnidirectional wheel design. One of the underlying ideas of this proposal is to reduce the high cost required to manufacture the different pieces and parts of the wheel. The analysis of this problem led to the proposal shown in Figure 5b. The manufacturing complexity of the optimal omnidirectional wheel of Figure 5a can be summarized in the intricate design of the piece that supports the axis of the free rollers and the in the difficulty of placing two bearings inside the optimum free rollers. The solution proposed in this paper to reduce the manufacturing complexity is to use inline skating wheels as free rollers (Figure 5b) and the creation of a compact rim using 3D printing. The use of these skate wheels as free rollers has the disadvantage of having a large gap between wheels, an aspect that will generate heavy impacts with the floor as a consequence of rotating the suboptimal omnidirectional wheel. Then, the question that arises is whether the passive suspension system of the APR-02 prototype will be able to avoid the propagation of the vibrations generated by these impacts to the mobile robot structure.

The main advantages of the selection of skating wheels are their huge commercial availability and reduced cost, the possibility to have covers with different hardness (74A (softer), 82A, 90A, 100A (harder)), the possibility to have different diameters (56, 63, 72, and 110 mm), the use of an internal ball bearing that facilitates the rotation of the skating wheel, and the hard and heavy outdoor usage originally expected for this kind of wheel. The advantages of using a rim based on 3D printing are the simplification of the manufacturing procedure and the reduction of the manufacturing cost. However, the most important advantage of 3D printing is the capability to create mechanical pieces with intricate internal and external shapes and holes that, in most cases, are very difficult to reproduce with CNC or plastic injection.

**Figure 6:**
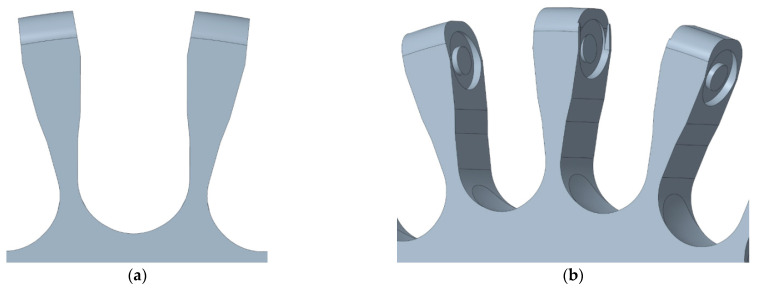
Detail of the rim of the suboptimal omnidirectional wheel: (**a**) detail of the clamps and (**b**) detail of the circular slots used to retain the skate wheels. The central clamp has an additional clearance in order to facilitate the insertion of the last skate wheel during assembly.

Figure 5b shows a partial CAD image covering the main parts of the suboptimal omnidirectional wheel proposed in this paper. The omnidirectional performances of the wheel are provided by 23 skate wheels used as transversal passive rollers. We selected the most common, easy to achieve, and cheap skate wheels: diameter 63 mm, hardness 82A. All the skate wheels have a hollow inner shaft that simplifies the support of the wheel to a fixed structure. Figure 6a shows a detail of the shape of the clamps proposed to support the skate wheels. Figure 6b shows the inner part of the clamps that have a circular slot to fit in and retain the axes of the rollers; one of the clamps is thinner and has an additional clearance in order to facilitate the entry of the last skate wheel attached to the rim. The shape of the clamps has free space to allow the rotation of the skate wheels without getting blocked by small objects. The axis of the DC motor of the mobile robot is also fitted in the center of the rim and locked with a screw.

### 4.3. Suboptimal Omnidirectional Wheel Implementation with 3D Printing

The implementation of the first prototypes of the suboptimal omnidirectional wheels with the FFF Printersys 325 3D printer was performed using Polylactic Acid (PLA) as a printing material and considering two infill densities. PLA was initially selected as a cheap printing material ideal for the trial and error procedure required to refine the implementation of the omnidirectional wheel. We expected to use strong printing materials such as PLA3D850 [14] in the definitive prototype implementation of the suboptimal omnidirectional wheel; however, the PLA implementation offered strong and convincing prototype results and was finally used as the 3D printing material used in the comparative implementation and analysis performed in this paper.

**Figure 7:**
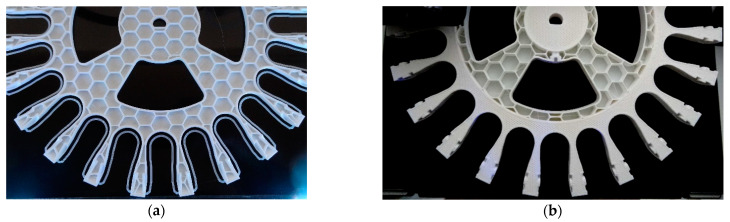
Detail of the printing procedure of the rim of the suboptimal omnidirectional wheel: (**a**) soft rim, with all the piece printed with a 10% honeycomb infill density, and (**b**) hard rim, with the radial inner and outer parts of the piece printed with a 100% honeycomb infill density and the intermediate radial part printed with a 10% honeycomb infill density.

Figure 7 shows an intermediate image obtained during the 3D printing of the rim. The design of the rim was adjusted to a diameter of 245 mm in order to fit in the 250 × 250 mm surface available in the 3D printer. Two 3D printing configurations were tested in this paper, labeled as soft rim (Figure 7a) and hard rim (Figure 7b). The common configurations used to print these two alternatives were a nozzle diameter of 0.4 mm, a layer height of 0.2 mm, the use of 3 outer walls, 3 top and bottom solid layers, an internal infill pattern called “full honeycomb” available in Simplify3D, a nozzle temperature of 215 °C, and a bed temperature of 50 °C.

Figure 7a shows the internal structure of the soft implementation of the compact rim proposed for the development of the suboptimal omnidirectional wheel. The specific 3D printing configuration of this soft rim was the use of a honeycomb internal structure with an infill density of only 10%. This soft rim was printed in 6 h 58 min and using 121.34 g of PLA. This printing configuration produces almost empty pieces with very low density and printing material usage. The combination of this low infill and the honeycomb internal support structure has the advantage of increasing the flexibility of large pieces. The final weight of the complete suboptimal omnidirectional wheel based on a soft rim was 1.773 kg, which represented a reduction of 33% in the weight relative to the optimal wheel implemented in aluminum. Moreover, the total number of pieces included in one complete omnidirectional wheel was reduced by 56%: 23 commercial low-cost skate wheels, one rim, and one screw, and the manufacturing cost was reduced one order of magnitude. In this case, the insertion of the skate wheels in this soft rim was simple thanks to the flexibility of the clamps. The suboptimal omnidirectional wheel obtained with this soft rim had a solid appearance because the skate wheels provided additional transversal resistance to the clamps. In the next sections we analyze whether this flexibility contributes to the reduction of the propagation of vibrations in the mobile robot.

Figure 7b shows the internal structure of the hard implementation of the compact rim proposed for the development of the suboptimal omnidirectional wheel. The 3D printing of this piece required additional procedures to divide the rim into three pieces in order to be able to apply a specific 3D printing configuration to each part of the rim. The external radial part and the inner radial part of the rim were printed with a honeycomb internal structure with an infill density of 100%. The intermediate radial part of the rim was configured with a honeycomb internal structure with an infill density of 10%. These three pieces needed to be joined by the slicer in order to create a final unique piece. This hard rim was printed in 15 h 26 min and using 216.39 g of PLA; just as an observation, these values increase to 23 h 11 min and using 294.40 g of PLA if printing all the rim with an infill density of 100%. This combined printing configuration produces very solid and strong pieces that do not have flexibility. In this case, the insertion of the skate wheels in this rim required more pressure and the insertion of the last skate wheel required an additional insertion clearance space (Figure 7b), otherwise the insertion of the las skate wheel would not be possible because of the rigidity of the clamps. The suboptimal omnidirectional wheel obtained with this hard rim had a strong and solid appearance. The final weight of the complete suboptimal omnidirectional wheel based on this hard rim was 1.868 kg, which represented a reduction of 30% in the weight relative to the optimal wheel implemented in aluminum and an increase of 95 g relative to the suboptimal wheel using a soft rim.

Finally, a destructive experiment showed that the direct force required to blend the thin part of the clamps was 6.2 kg for the soft rim and 8.6 kg for the hard rim. The insertion of the skate wheels in the rim introduced additional transversal resistance that distributed any force along the rim, so we did not expect the generation of transversal forces high enough to blend or break the thinner part of the clamp. It was the opposite of what happened with the optimal omnidirectional wheel in which the thinner part of the support piece had to support up to the 33% of the weight of the mobile robot, forcing the use of durable aluminum in the final implementation of the optimal wheel.

## 5. Comparing the Individual Performances of One Omnidirectional Wheel

This section compares the individual performances of one omnidirectional wheel by testing the vibrations generated by one omnidirectional wheel. This experimental configuration simplifies the experimentation with only one omnidirectional wheel but does not summarize all the effects generated during a motion. In this case the acceleration patterns were measured by placing the measurement module over the DC motor of the omnidirectional wheel and in the location of point A of the mobile robot. In this comparative experiment the measurement module was configured to apply power only to the omnidirectional wheel being tested. Then the measurement module applied power to one DC motor, measured the rotational speed of the motor, and measured the accelerations generated during the experiment. The application of different PWM simulated different operation conditions of the omnidirectional wheel.

**Figure 8:**
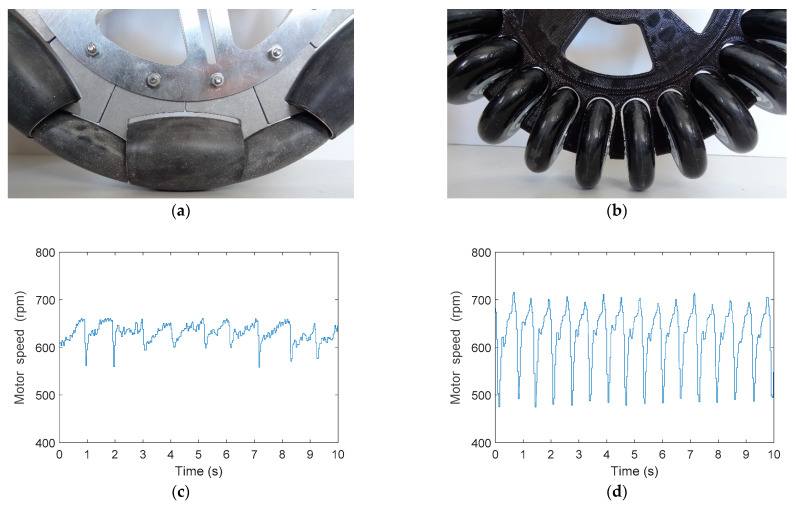
(**a**) Detail of the optimal omnidirectional wheel in contact with the floor, (**b**) detail of the suboptimal omnidirectional wheel in contact with the floor; (**c**) evolution of the rotational speed of one DC motor driving the optimal omnidirectional wheel shown in (**a**), and (**d**) evolution of the rotational speed of one DC motor driving the suboptimal omnidirectional wheel shown in (**b**).

Figure 8 shows the optimal and suboptimal wheel and the evolution of the rotational speed directly measured by the encoder of the DC motor attached to the wheel in the case of very low rotation speed (20% PWM) and no control loop applied to the DC motor. The evolution of the rotational speed revealed the main differences of the optimal and the suboptimal wheels and the effect that generated the vibrations transferred to the structure of the mobile robot. However, this difference in the evolution of the rotational speed was not easy measurable at higher rotation speeds because of the inertia of the wheel and the low resolution of the encoder. Figure 8a shows a detail of the optimal omnidirectional wheel in contact with the floor and Figure 8c the evolution of the rotational speed of the DC motor using this optimal omnidirectional wheel. In this case, the minimum gap of the optimal omnidirectional slightly affected the rotation of the DC motor. Alternatively, Figure 8b shows a detail of the suboptimal omnidirectional wheel in contact with the floor and Figure 8d shows that the huge gap between the skate wheels had a strong influence on the rotation of the DC motor that drives the wheel. This periodic reduction of the rotational speed was alternative evidence that confirmed that the suboptimal omnidirectional wheel generated more vibrations than the optimal implementation due to the effect of the impact of the free rollers with the floor.

**Figure 9:**
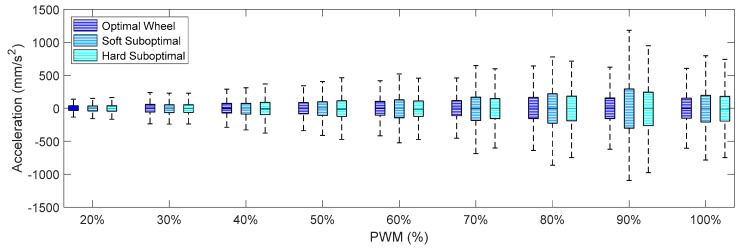
Amplitude of the accelerations measured on the X-axis for different PWM percentages when using the optimal and suboptimal omnidirectional wheels.

**Figure 10:**
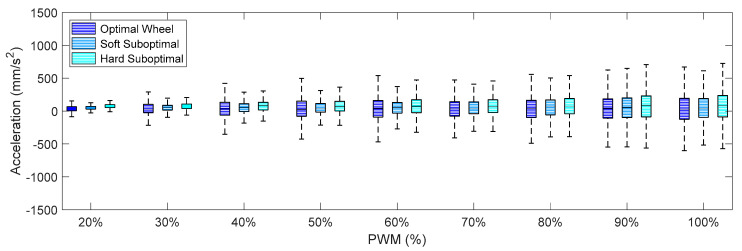
Amplitude of the accelerations measured on the Y-axis for different PWM percentages when using the optimal and suboptimal omnidirectional wheels.

**Figure 11:**
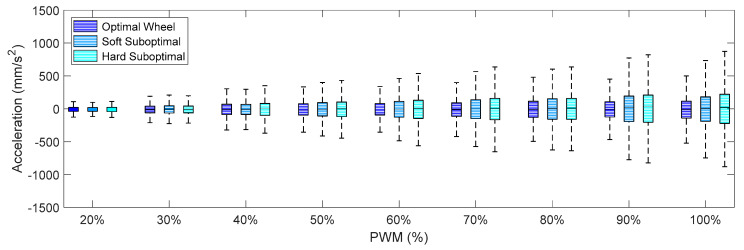
Amplitude of the accelerations measured on the Z-axis for different PWM percentages when using the optimal and suboptimal omnidirectional wheels.

Figure 9, Figure 10 and Figure 11 summarize the X-axis, Y-axis, and Z-axis acceleration measured directly on the motor that drives the omnidirectional wheel. These figures show that (1) the IQR of the acceleration patterns increased as the PWM and the rotational speed of the DC motor increased, and (2) the IQR of the acceleration patters obtained using the suboptimal omnidirectional wheels in a range of a PWM of up to 60% were similar or slightly higher when using the optimal omnidirectional wheel. Figure 9 shows that the IQR ratio between the two wheels in the X-axis increased up to 100% for a PWM of 90%. However, a PWM value of 60% is a reference that is equivalent to the nominal forward speed used in the mobile robot and is not usually exceeded in a normal operation.

**Figure 12:**
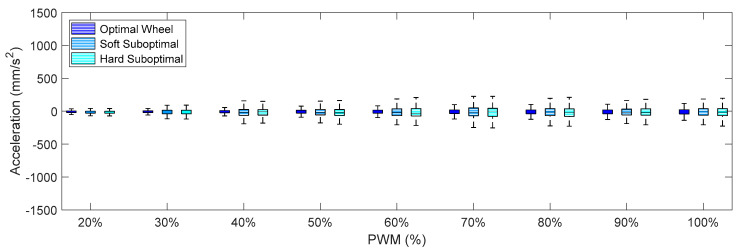
Amplitude of the accelerations measured on the X-axis for different PWM percentages comparing the three different omnidirectional wheels and the effect of the passive suspension system.

**Figure 13:**
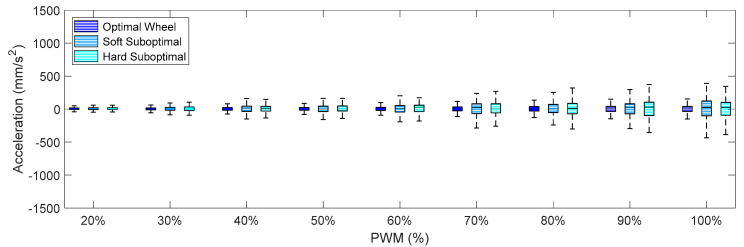
Amplitude of the accelerations measured on the on Y-axis for different PWM percentages comparing the three different omnidirectional wheels and the effect of the passive suspension system.

**Figure 14:**
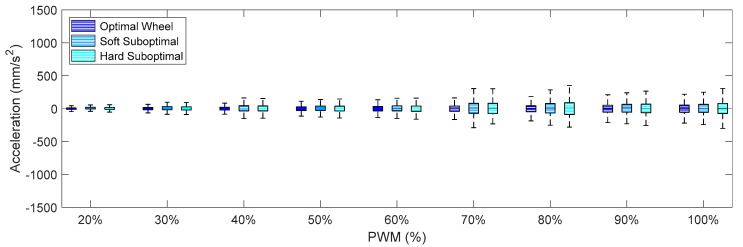
Amplitude of the accelerations measured on the Z-axis for different PWM percentages comparing the three different omnidirectional wheels and the effect of the passive suspension system.

Finally, Figure 12, Figure 13 and Figure 14 show the X-axis, Y-axis, and Z-axis accelerations measured on point A when only one motor of the omnidirectional wheels was powered. These figures show that the reduction of the vibrations originated with the passive suspension system, which was significantly reduced in all axes.

Comparing the IQR of the acceleration shown in Figure 9, Figure 10, Figure 11, Figure 12, Figure 13 and Figure 14, the general conclusion that arises is that the soft suboptimal omnidirectional wheel produced similar or worse IQR than the hard suboptimal omnidirectional wheel. Results show that the flexibility of the soft rim did not contribute to reduce the transmission of vibrations from the wheel to the structure of the mobile robot, especially in the Z-axis (Figure 14). A closer inspection of the rotation of the soft suboptimal omnidirectional wheel revealed an asymmetric deformation of the wheel that contributed to a worse transmission of vibrations to the structure of the mobile robot. We repeated these experiments with different wheels in order to discard the coincidence of having had printing problems or having used a corrupted PLA but the results were the same in all experiments. After discarding many hypotheses we found the motivation for this asymmetric deformation during a rotation in the image of Figure 7a. This image shows that the internal honeycomb structure had no radial symmetry, meaning that the different clamps of the rim had a different internal honeycomb support structure. Therefore, the resistance to deformation of the clamps was not constant and produced an asymmetric deformation of the soft rim during a rotation. This problem cannot be solved easily by changing the infill pattern or the slicer program because none of the standard infill patterns provide radial symmetry. At this moment, the solution to this problem requires the redesign of the soft rim as an empty piece and the incorporation of a custom radial infill pattern, but we discarded this alternative as it may require additional experimental validation of the improvement. Therefore, as an alternative to the optimal omnidirectional wheel used in the APR mobile robots [7,22], we propose the use of a suboptimal omnidirectional wheel based on the use of a hard rim created by combining a honeycomb infill patter with a 100% infill density in the inner and outer radial parts of the rim and a honeycomb infill pattern with a 10% infill density in the middle part of the rim. 

## 6. Evaluation of Mobile Robot APR-02 with the Suboptimal Omnidirectional Wheels

**Figure 15:**
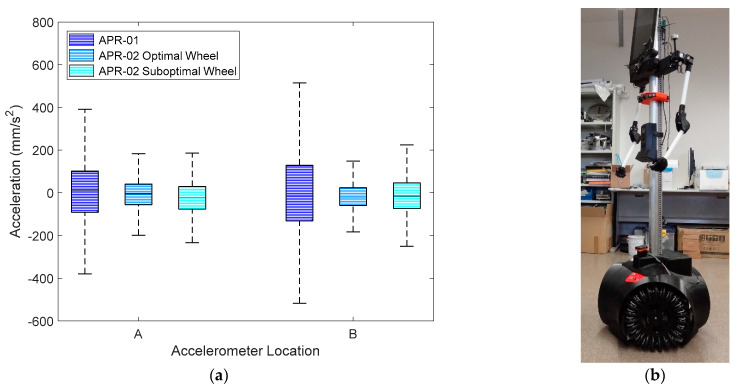
(**a**) Representation of the amplitude of the accelerations measured on the X-axis at location A and B for the APR-01, APR-02 with the optimal omnidirectional wheel, and APR-02 with the proposed suboptimal omnidirectional wheel, and (**b**) image of APR-03 using the suboptimal omnidirectional wheels.

This section shows the final comparative implementation of the suboptimal omnidirectional wheel design proposed in this paper. This final experiment consisted of the measurement of the amplitude of the accelerations at locations A (base) and B (head) of APR-02 using the proposed suboptimal omnidirectional wheel and a forward velocity equivalent to the application of a PWM of 60% in two of the DC motors. Figure 15a repeats the information in Figure 4 with the inclusion of the new results obtained in this experiment. The largest acceleration was obtained again on the X-axis, longitudinal to the forward displacement of the mobile robot. Figure 15a shows that the IQR of the acceleration measured in the APR-02 mobile robots at location A was almost the same when using the optimal and the suboptimal wheel. The IQR of the acceleration measured in point B of the APR02 with the suboptimal omnidirectional wheel was 54% lower than APR-01 and 43% higher than the APR-02 using the original optimal omnidirectional wheel. These acceleration levels were not visually perceived as a problem and did not compromise the practical operation of the mobile robot using the suboptimal omnidirectional wheel. Figure 15b shows an image of mobile robot APR-03 (a clone of APR-02) used to additionally validate the results obtained with the suboptimal omnidirectional wheels proposed in this paper.

**Figure 16:**
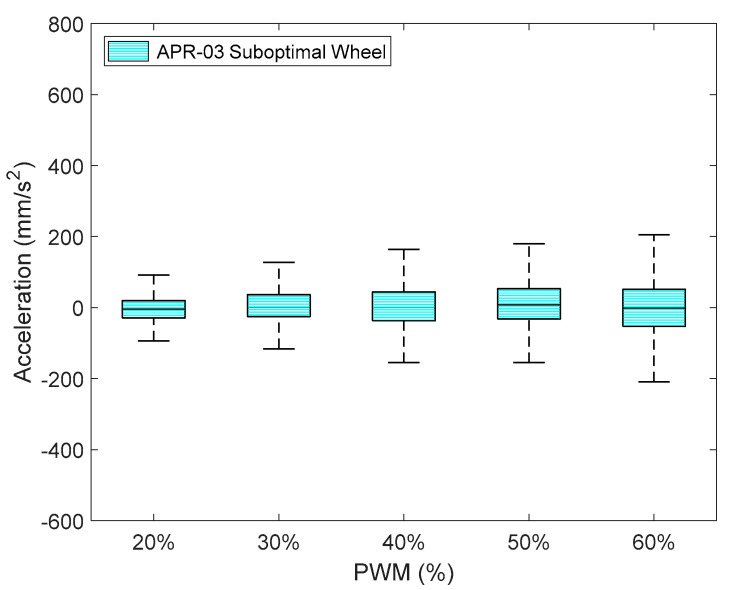
Representation of the X-axis amplitude of the acceleration measured at point B of APR-03 using the suboptimal omnidirectional wheels with different PWM applied to two DC motors of the omnidirectional motion system.

Finally, Figure 16 details the IQR of the amplitude of the acceleration measured at point B (head) of the APR-03 using the suboptimal omnidirectional wheels and different PWM. In this case the PWM value represents the PWM level applied to the two frontal DC motors of the APR-03 mobile robot, and the third motor was electrically braked in order to generate a forward displacement. These final results obtained with this alternative APR-03 mobile robot prototype confirmed the results obtained with the APR-02. Figure 16 shows that the IQR of the acceleration obtained with the APR-03 robot prototype for a PWM of 60% was almost the same as that obtained with the APR-02 prototype. These results also show that the IQR linearly decreased if the PWM applied was lower than this 60% nominal reference value.

## 7. Discussion and Conclusions

This paper empirically demonstrated that the APR omnidirectional mobile robots can take advantage of their passive suspension system in order to use both optimal omnidirectional wheels and suboptimal omnidirectional wheels. The optimal omnidirectional wheels were originally manufactured in aluminum and CNC and the suboptimal omnidirectional wheel proposed is based on the combined use of commercial low-cost skate wheels designed for heavy usage and 3D printing, which allows for the creation of complex shapes and geometries with hollow parts and different infill patterns and densities.

On the one hand, the main advantage of an optimal omnidirectional wheel is the use of the minimum inner gap between the free rollers, and the disadvantages are its high manufacturing cost and its poor performance on a floor with small obstacles. On the other hand, the main disadvantage of a suboptimal omnidirectional wheel is that a larger gap between rollers produces a greater impact force with the floor, generating more vibrations as a consequence of the rotation of the wheels. However, the main comparative advantages of the suboptimal omnidirectional wheel proposed in this paper are its low manufacturing cost and the possibility of taking advantage of the gap to pass over small objects or wires that usually stop a mobile robot using optimal omnidirectional wheels. Additionally, the toothed shape of the suboptimal omnidirectional wheel opens the possibility of providing traction outdoors and even on unpaved surfaces. 

This paper compared the vibrations generated by two prototype assistant personal robots: APR-01 using a three-wheel omnidirectional motion system and APR-02 using a three wheel omnidirectional motion system and a suspension system. Additionally, an APR-03 prototype, which is a clone of APR-02, was used to validate the results obtained with APR-02. The experimental results obtained with mobile robots APR-01 without a suspension system and APR-02 with a passive suspension system validated the results of Moreno et al. [21] and confirmed that the use of a passive suspension system largely reduces the transmission of vibrations from the wheels to the head of this heavy-weighted and tall omnidirectional mobile robot.

The experimental results also showed that the acceleration measured in the base of APR-02 (measurement point A) moving forward at its nominal velocity were almost the same when using the optimal and the suboptimal omnidirectional wheels. In the same case, the acceleration measured in the head of APR-02 (measurement point B) was 43% higher because of the use of the suboptimal omnidirectional wheels, although this vibration levels were not visually perceived and did not compromise the practical application of the mobile robot using the suboptimal omnidirectional wheel. Comparatively, the APR-02 mobile robot with suboptimal omnidirectional wheels had better performance than the APR-01 mobile robot prototype presented by Clotet et al. [22]. In this case the APR-02 mobile robot moving forward at its nominal velocity reduced the acceleration levels measured in the APR-01 by 54%, and the cost of manufacturing the omnidirectional wheels was reduced one order of magnitude. These improvements were additionally validated using a third mobile robot prototype with the same passive suspension and obtaining similar results.

Future work will be focused on the development of new mobile platforms optimized to take full advantage of the omnidirectional motion system in unstructured environments, and on the evaluation of the proposed suboptimal omnidirectional wheel on rough indoor and outdoor floors.

## Data Availability

Data sharing not applicable.

## Supplementary Materials

The dataset used in this paper is available online as a supplementary material. The following are available online at https://www.mdpi.com/1424-8220/21/3/865/s1.
